# A Recombinant Raccoon Poxvirus Vaccine Expressing both *Yersinia pestis* F1 and Truncated V Antigens Protects Animals against Lethal Plague

**DOI:** 10.3390/vaccines2040772

**Published:** 2014-10-27

**Authors:** Tonie E. Rocke, Brock Kingstad-Bakke, Willy Berlier, Jorge E. Osorio

**Affiliations:** 1 National Wildlife Health Center, U.S. Geological Survey, 6006 Schroeder Rd., Madison, WI 53711, USA; 2 Department of Pathobiological Sciences, School of Veterinary Medicine, University of Wisconsin, Madison, WI 53706, USA; E-Mails: bunshi@gmail.com (B.K.-B.); willyberlier@gmail.com (W.B.); osorio@svm.vetmed.wisc.edu (J.E.O.)

**Keywords:** raccoon poxvirus, *Yersinia pestis*, plague, vaccine, prairie dogs

## Abstract

In previous studies, we demonstrated in mice and prairie dogs that simultaneous administration of two recombinant raccoon poxviruses (*r*RCN) expressing *Yersinia pestis* antigens (F1 and V307—a truncated version of the V protein) provided superior protection against plague challenge compared to individual single antigen constructs. To reduce costs of vaccine production and facilitate implementation of a sylvatic plague vaccine (SPV) control program for prairie dogs, a dual antigen construct is more desirable. Here we report the construction and characterization of a novel RCN-vectored vaccine that simultaneously expresses both F1 and V307 antigens. This dual antigen vaccine provided similar levels of protection against plague in both mice and prairie dogs as compared to simultaneous administration of the two single antigen constructs and was also shown to protect mice against an F1 negative strain of *Y. pestis.* The equivalent safety, immunogenicity and efficacy profile of the dual RCN-F1/V307 construct warrants further evaluation in field efficacy studies in sylvatic plague endemic areas.

## 1. Introduction

Plague, caused by the bacterium, *Yersinia pestis*, is a zoonotic disease of wild rodents that has severely impacted mammalian populations since its introduction into North America in the early 1900s [1]. In fact, the disease probably contributed to the demise of several endangered species or at least hinders their recovery [2], including the black-footed ferret (*Mustela nigripes*) and the Utah prairie dog (*Cynomys parvidens*). Although plague in humans is rare in the U.S. (3–10 cases per year), its occurrence causes alarm, and outbreaks in rodents can result in park closures or curtailment of other human activities. For these reasons, methods to manage sylvatic plague are highly desired. Although pesticides are effective in reducing fleas that transmit the disease, their application is labor intensive and costly, and the effects of the general insecticides used (e.g., deltamethrin) are indiscriminate, killing other arthropods. As an alternative, we have developed a highly efficacious, orally-delivered sylvatic plague vaccine (SPV) that protects prairie dogs against plague challenge.

Our approach is similar to the successful oral vaccination program for rabies in European and North American carnivores that utilizes vaccinia as a vector for rabies glycoprotein [3,4]. Using a similar orthopoxvirus, raccoonpox (RCN), we have designed and tested several vaccine constructs that express antigens known to be protective against plague [5,6]. Our first vaccine construct expressed the F1 capsular antigen (designated RCN-F1) and was shown to be protective against plague in both laboratory mice [5] and prairie dogs [7]. Due to the occurrence of F1 negative strains of *Y. pestis*, we designed a second vaccine construct expressing a truncated version of the *lcr*V gene (herein designated RCN-V307). Studies confirmed the protective efficacy of truncated V constructs against F1 negative strains [8] and further demonstrated that combined administration of RCN-F1 and RCN-V307 increased protection against plague in both mice [9] and prairie dogs [6]. However, for a large-scale vaccination program, a single vaccine construct expressing both antigens is more desirable as it would significantly reduce costs of production.

Here, we describe the construction of an RCN-vectored vaccine expressing both F1 and V307 antigens (designated RCN-F1/V307). The efficacy of RCN-F1/V307 was compared to that of RCN-F1, RCN-V307, and combined RCN-F1 + RCN-V307 in a mouse challenge model that included both F1 positive and F1 negative strains of *Y. pestis*. We also evaluated plague protection in prairie dogs that were orally vaccinated after consuming baits containing the dual antigen construct.

## 2. Experimental

### 2.1. Cells and Viruses

Rat embryonic fibroblasts [Rat-2 (ATCC #CRL-1764)] and African green monkey kidney epithelial [BSC-1 (ATCC #CCL-26) and Vero (ATCC#CCL-18)] cells were maintained at 37 °C and 5% CO_2_ in M199 medium supplemented with 0.01 g/L L-glutamine and 5% fetal bovine serum (FBS) and were used for culturing virus. Raccoon poxvirus (RCN) Herman strain [10] was mixed 1:1 with trypsin-versene solution (0.05% trypsin; 0.02% EDTA in Earle’s Balanced Salt Solution) and incubated for 15 min at 37 °C prior to inoculation onto cells.

### 2.2. Construction of pTK Transfer Vectors

**Figure 1:**
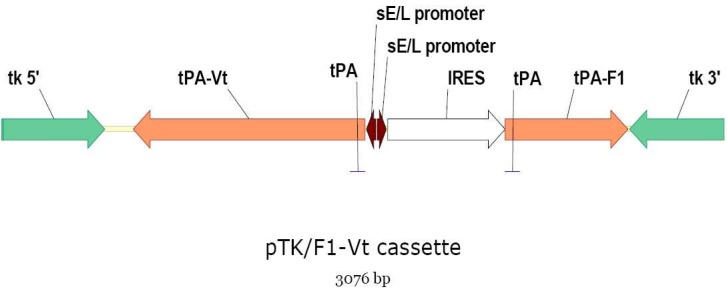
Schematic of the plasmid (pTK) used to produce the dual antigen plague vaccine construct, raccoon poxvirus expressing F1 and V antigens (RCN-F1/V307). The internal ribosomal entry site (IRES) was used as a translation enhancer for the F1 gene. The F1 and V genes were fused to the tissue plasminogen activator (tPA) secretory signal.

Construction of RCN-F1 and RCN-V307 viruses was described previously [5,9]. For the RCN-F1/V307 construct, both F1 and *lcr*V (V307, coding for a 307-aa C-terminally truncated V antigen) genes from *Y. pestis* were cloned into the pTK shuttle vector, so that the introduced genes and upstream *cis*-acting elements were flanked by RCN thymidine kinase gene (*tk*) sequences (Figure 1). The internal ribosomal entry site (IRES) from encephalomyocarditis virus (EMCV-IRES) preceding the V307 gene was removed to avoid the interferences observed when two IRES are present in the same construct. In an attempt to increase antigen expression, the synthetic strong early/late promoter (sE/L) was used to express both F1 and V307 genes. The tissue plasminogen activator (tPA) secretory signal sequence was inserted in-frame before both F1 and V307 genes (Figure 1).

### 2.3. Generation of Recombinant RCN Constructs

The *Y. pestis* genes were inserted into the RCN genome by homologous recombination as previously described [5]. Briefly, BSC-1 cells were infected with wild type RCN and transfected with the pTK shuttle vector. Three days post-infection/transfection, cells were harvested and the lysate was re-plated on Rat-2 cells in medium containing 5-bromo-2-deoxyuridine (BrdU) for selection of TK-RCNV. Purified viral clones were then amplified for large scale purification in Vero cells as described previously [5] and passaged 10 times to confirm the stability of the insert using PCR. *In vitro* expression was determined by western blot as described previously, using polyclonal mouse anti-V and polyclonal rabbit anti-F1 antibodies, secondary antibodies conjugated to alkaline-phosphatase (KPL Inc., Gaithersburg, MD, USA) and BCIP/NBT revelation (KPL Inc.).

### 2.4. Mouse Vaccination and Challenge Studies

All animal studies were conducted at the USGS National Wildlife Health Center (NWHC) in accordance with NWHC’s animal care and use committee (NWHC #EP050614-A1 and #EP100421). First, we compared the protective efficacy of the *r*RCN single and dual antigen constructs in mice. Five groups of twenty-four A/J mice (4 week-old) received one of the following treatments: RCN-F1, RCN-V307, a simultaneous injection of RCN-F1 and RCN-V307, the RCN-F1/V307 dual construct or PBS (negative control). Treatments were injected intramuscularly (i.m.) in the thigh with a dose of 5 × 10^7^ pfu in 0.1 mL. Animals that received two *r*RCN constructs were inoculated with each virus in separate thighs. All groups received a boost vaccination (same formulations/dosage/route) four weeks later. To determine the kinetics of vaccine immune responses and protection, six animals in each group were challenged at 7, 14, 28, and 56 days post booster vaccination (dpb), with *Y. pestis* (CO92 isolate, provided by U.S. Army Medical Research Institute of Infectious Diseases-USAMRIID) at a dose of 1.4 × 10^4^ mouse LD_50_s by intradermal (i.d.) injection in the abdominal region. The mice were monitored for 21 days for signs of illness or death. Animals with obvious clinical signs of plague (labored breathing, severe lethargy, *etc.*) were humanely euthanized.

To confirm the efficacy of the dual antigen construct against varying doses of plague, groups of six A/J mice each were vaccinated with RCN-F1/V307, same dose and route as described above, and boosted four weeks later. Three groups of four mice each served as controls. The mice were all bled on 21 dpb and each group of vaccine-treated and control mice were challenged as above with varying doses of *Y. pestis* CO92 strain (1.4 × 10^3^, 1.4 × 10^4^, or 8.0 × 10^5^ mouse LD_50_s).

The third study examined the efficacy of the RCN-F1/V307 dual antigen construct against a *Y. pestis* F1 negative strain. Three groups of 24 A/J mice were vaccinated and boosted with either RCN-F1, simultaneous injections of RCN-F1 and RCN-V307, or RCN-F1/V307 as described above. At 21 and 42 dpb, 6 animals in each group were challenged as above with an F1 negative strain of *Y. pestis* (Java 9, YERS022, provided by USAMRIID) at a dose of 1.4 × 10^4^ or 1.4 × 10^5^ LD_50_s.

### 2.5. Prairie Dog Oral Vaccination and Challenge Study

Finally, we evaluated the efficacy of the RCN-F1/V307 dual antigen construct delivered via edible baits in prairie dogs (PDs), our primary target animal. Adult black-tailed prairie dogs (*Cynomys ludovicianus*) were captured from wild colonies near Wall, SD, USA (43.992N, 102.241W) and dusted with carbaryl prior to shipment to NWHC. Upon arrival, they were inspected for external parasites (none found), injected with an anthelminthic (200 μg/kg of Ivermectin, Merck & Co., Inc, West Point, PA, USA), then treated with Advantage flea control (Imidacloprid; Bayer HealthCare, Shawnee Mission, KS) and inserted with micropchips (Avid Identification Systems, Inc., Folsom, LA, USA) to identify individuals. Blood was also drawn upon arrival to test for antibodies to plague antigens as described below, and the animals were group-housed and fed as previously described [6]. The animals were randomly assigned to one of five treatment groups and housed in separate rooms.

RCN constructs (RCN-F1/V307 or RCN-TK-) were mixed into 4 g baits and offered to PDs for voluntary consumption. Three groups were fed baits containing RCN-F1/V307; one of these groups (group 4) also received baits a second time at 240 days post initial vaccination (dpv). Two control groups received baits containing the empty vector (RCN-TK-) as a placebo, designated groups 1a and 1b. The bait formulation was supplied by a commercial source (Food Source Lures, Alabaster, AL, USA) and peanut butter was added to increase palatability. To increase vaccine or placebo intake by PDs, fresh vegetables were withheld for 48 h prior to vaccination and food pellets were withheld for 12–18 h prior to vaccination. Animals were individually placed in pet carriers with either one vaccine-laden bait or one placebo bait. After 2–4 h, animals were released back into their animal rooms, and bait consumption was recorded. This process was performed again the next day, so that all animals were offered two baits. Group 4 animals, that received vaccine baits a second time, were treated similarly. Animals that did not eat any baits were removed from the study (one animal).

Blood samples were drawn from prairie dogs at approximately 30 dpv (*n* = 70), 180 dpv (*n* = 41) and 270 dpv (*n* = 41). At 30 dpv, one vaccine-treated (group 2) and one placebo group (group 1a) were challenged with *Y. pestis*. On 270 dpv, the remaining three groups were challenged: one vaccine-treated group (group 3), one vaccine treated group that consumed baits a second time 240 dpv (group 4), and the remaining placebo group (1b). PDs were challenged with *Y. pestis* (CO92) at a dosage of 3500 mouse LD_50_s via subcutaneous injection in the right hip region. They were monitored 2–3 times daily for 28 days for signs of illness or death. Carcasses were removed immediately upon discovery and stored at −20 °C until processed. Animals with obvious clinical signs (labored breathing, disinclination to move) were humanely euthanized, as were all survivors at the end of the 28-day period. Tissue samples for bacteriology were aseptically collected and stored at −20 °C, and plague was confirmed as the cause of death in selected animals.

### 2.6. Serology

Blood samples were collected from the medial saphenous vein of mice and PDs. Sera were collected and stored at −20 °C. Antibody titers (total IgG, IgG1, and IgG2c for mice and IgG only for PDs) to *Y. pestis* F1 and V were determined by ELISA [5,11]. Serum samples were serially diluted 4-fold from 1:160 to 1:163,840 and tested in duplicate. Titers ≤1:160 were considered negative and titers <1:160 were designated as 1:40 for analyses. The highest dilution that was positive (exceeded the mean of four negative control samples by three standard deviations) was considered the endpoint and its reciprocal value recorded as the titer.

### 2.7. Statistical Analyses

All analyses were performed with SAS statistical software (SAS Institute Inc., Cary NC, USA). The effects of treatments and antibody titers on survival rates were evaluated with the Cox proportional hazards model, and PD survival curves were generated using Kaplan Meier analysis. Antibody titers were log-transformed prior to analysis. Pre-challenge geometric anti-F1 and anti-V titers and interactions between the two were analyzed in relation to time to death using the Cox proportional hazards model for the first and third mouse experiments. Data from all mouse experiments were combined and treatment groups were compared using analysis of variance. Likewise, antibody titers of PDs consuming vaccine baits were combined and compared at each time point using analysis of variance.

## 3. Results

### 3.1. In Vitro Characterization of RCN-F1/V307 Construct

**Figure 2:**
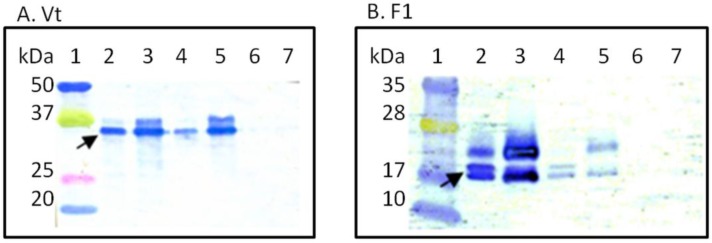
Western blot analysis of dual antigen plague vaccine construct, RCN-F1/V307, versus individual constructs, RCN-F1 and RCN-V307. Vero cell monolayers were infected with RCN constructs at an MOI of 1 and pellet (pt) and supernatant (sup) fractions were harvested 24 h post infection. Blot A was probed with anti-V antibody, and blot B was probed with anti-F1 antibody. Arrows indicate Vt and F1 proteins. Lanes for blot A are: (1) ladder, (2) RCN-V307 pt, (3) RCN-V307 sup, (4) RCN-F1/V307 pt, (5) RCN-F1/V307 sup, (6) RCN-F1 pt, (7) RCN-F1 sup. Lanes for Blot B are: (1) ladder, (2) RCN-F1 pt, (3) RCN-F1 sup, (4) RCN-F1/V307 pt, (5) RCN-F1/V307 sup, (6) RCN-V307 pt, (7) RCN-V307 sup.

A dual antigen RCN-vectored vaccine was successfully constructed containing both F1 and a truncated form of the lcrV gene (V307). The genetic stability of the RCN-F1/V307 construct was assessed following ten blind passages in Vero cells. No changes in PCR fragment size and sequence were observed for the F1 and V307 inserts [12]. The *in vitro* expression of RCN-F1/V307 was examined by western blot analyses at 24 h PI in comparison to RCN-F1 and RCN-V307 (Figure 2). Samples were treated identically and to ensure equal loading, the same samples were used for both blots A and B. No differences were detected in the migration between proteins expressed from the RCN-F1/V307 construct and the individual RCN-F1 and RCN-V307 constructs. For the RCN-F1/V307 construct, *Y. pestis* antigens were detected in the cell pellet and supernatant of Vero-infected cells. Similar levels of V307 expression were observed in the RCN-V307 and RCN-F1/V307 constructs. In contrast, F1 expression was significantly lower in the RCN-F1/V307 than the RCN-F1 construct. Multiple-sized fragments are due to both glycosylated and non-glycosylated forms of the protein being expressed [8].

### 3.2. Mouse Vaccination and Challenge Studies

The first mouse experiment compared the protective efficacy and immune responses of all rRCN vaccine constructs at different days post boost (dpb) vaccination. Plague challenge (1.4 × 10^4^ mouse LD_50_s) on day 7 dpb resulted in poor survival (17%–33%) in all groups. Protection increased significantly 14 and 28 dpb for all groups with the exception of those vaccinated with RCN-V307 alone (Table 1). The best protection (100%) was provided by the combination vaccine in animals challenged on days 14 and 28, but survival dropped to 33% on 56 dpb. RCN-F1/V307 provided the best survival (67%) in mice at 56 dpb, although risk ratios did not differ significantly (*p* > 0.05) from RCN-F1 or RCN-F1 + RCN-V307. Higher pre-challenge anti-F1 antibody titers (regardless of dpb) resulted in the lowest risk ratio (*p* < 0.0001). Anti-V antibody titers in the absence of anti-F1 were not significant (*p* = 0.2219), but the interaction of anti-F1 and anti-V was significant (*p* = 0.0086).

**Table 1:** Vaccine treatments administered to A/J mice via intramuscular inoculation and their response to challenge with *Yersinia pestis* (CO92) at various days post booster vaccination (dpb) at a dosage of 1.4 × 10^4^ mouse LD_50_s. Risk ratios are calculated in relation to RCN-F1/V307 at day 56 dpb. Lower and upper 95% indicate 95% confidence intervals on the risk ratio.

Treatment	DPB	% Survival	Risk Ratio	Prob > Chisq	Lower 95%	Upper 95%
Control	7	0	9.04	0.01	1.71	66.63
	14	0	4.15	0.09	0.80	30.34
	28	0	7.27	0.02	1.39	53.13
	56	0	4.31	0.08	0.84	31.24
RCN-F1	7	33	2.70	0.24	0.53	19.47
	14	50	1.56	0.62	0.26	11.83
	28	67	0.85	0.87	0.10	7.08
	56	50	1.46	0.67	0.24	11.11
RCN-V307	7	33	2.52	0.27	0.49	18.24
	14	17	2.88	0.18	0.61	20.23
	28	17	4.70	0.05	1.00	33.00
	56	0	5.90	0.02	1.33	40.88
RCN-F1 + RCN-V307	7	33	1.88	0.45	0.37	13.58
	14	100	0.00	0.06	0.00	1.15
	28	100	0.00	0.06	0.00	1.15
	56	33	1.87	0.46	0.37	13.50
RCN-F1/V307	7	17	3.78	0.09	0.81	26.48
	14	50	1.53	0.64	0.25	11.59
	28	67	0.80	0.82	0.10	6.67
	56	67	1.00			

The protective efficacy of RCN-F1/V307 was confirmed in the second experiment. Eighty-three % of vaccinated mice survived *Y. pestis* challenge at a dose of 1.4 × 10^4^ mouse LD_50_s and 50% survived challenge at an even higher dose, 8.0 × 10^5^ mouse LD_50_s, whereas all controls succumbed to infection within five days post challenge (Table 2).

**Table 2:** Survival rates of A/J mice vaccinated with RCN F1/Vt and challenged with *Y. pestis.*

Challenge Dose	% Survival (*n*) by Treatment	
	Control	RCN-F1/Vt
1.4 × 10^3^	0 (4)	80 (5)
1.4 × 10^4^	0 (4)	83 (6)
8.0 × 10^5^	0 (4)	50 (6)

In the third experiment, mice vaccinated with *r*RCN constructs were challenged with an F1-negative strain of *Y. pestis* at two different dosages (1.4 × 10^4^ or 1.4 × 10^5^ LD_50_s). As expected, all mice vaccinated with RCN-F1 alone succumbed to challenge within 3–7 days post challenge. Mice that received RCN-F1/V307 had higher survival rates (50%–67%) than mice that received RCN F1 + RCN-V307 (33%–50%) at both challenge dosages. Risk ratios were not significantly different (*p* > 0.05) between these treatment groups, except at the higher challenge dose at 21 dpb (Table 3). As expected, in this experiment, anti-V antibody titers were associated with the lowest risk ratio (*p* < 0.0001). Anti-F1 antibodies were insignificant (*p* = 0.42), as was the interaction between anti-F1 and anti-V antibodies (*p* = 0.80).

**Table 3:** Vaccine treatments administered to A/J mice via intramuscular inoculation and their response to challenge with two dosages (low—1.4 × 10^4^ mouse LD_50_s and high—1.4 × 10^5^ mouse LD_50_s) of an F1 negative strain of *Yersinia pestis* (Java 9, YERS022) at 21 and 42 days post booster vaccination (dpb). Risk ratios are calculated in relation to RCN F1/V307 at the high dose and 42 days dpb.

Treatment	Challenge Dose	DPB	% Survival	Risk Ratio	Prob > Chisq	Lower 95%	Upper 95%
RCN-F1	low	21	0	3.69	0.08	0.85	25.26
		42	0	4.59	0.04	1.04	31.77
	high	21	0	5.00	0.03	1.12	34.67
		42	0	9.70	0.00	2.16	67.82
RCN-F1 + RCN-V307	low	21	50	1.51	0.65	0.25	11.48
		42	33	2.36	0.31	0.46	17.02
	high	21	0	8.29	0.01	1.87	57.44
		42	50	1.69	0.56	0.28	12.84
RCN-F1/V307	low	21	50	1.56	0.62	0.26	11.84
		42	50	1.69	0.56	0.28	12.84
	high	21	50	1.44	0.68	0.24	10.96
		42	67	1.00			

The dual-antigen construct (RCN-F1/V307) and the combination vaccine (RCN-F1 + RCN-V307) induced different serological response patterns in mice. All pre-challenge antibody titers from the 3 mouse experiments were combined and presented in Table 4. Animals that received RCN-F1/V307 had very high anti-V titers and only moderate anti-F1 titers. In contrast, mice that received RCN-F1 and RCN-V307 in combination had high anti-F1 titers and only moderate anti-V titers. Mice that received RCN-V307 alone had low anti-V antibody titers (Table 4). Analysis of IgG subclasses for both antigens in all immunized animals showed a strong response for IgG1, with a lowered response to IgG2c [12].

**Table 4:** Pre-challenge anti-F1 and anti-V IgG geometric mean titers ± standard error (SE) for A/J/mice averaged for each vaccine treatment over all experiments.

Treatment	*n*	Mean Anti-F1 ± SE*	Mean Anti-V ± SE*
RCN-F1/V307	65	2.86 ± 0.09 ^C^	4.25 ± 0.08 ^A^
RCN-F1 + RCN-V307	48	4.14 ± 0.13 ^A^	3.09 ± 0.14 ^B^
RCN-V307	24	1.60 ± 0.00 ^D^	2.73 ± 0.15 ^C^
RCN-F1	48	3.80 ± 0.14 ^B^	1.60 ± 0.00 ^D^
Control	28	1.60 ± 0.00 ^D^	1.60 ± 0.00 ^D^

* Values that do not share a letter within columns are significantly different at *p* < 0.05.

### 3.3. Prairie Dog Vaccination and Challenge Study

**Figure 3:**
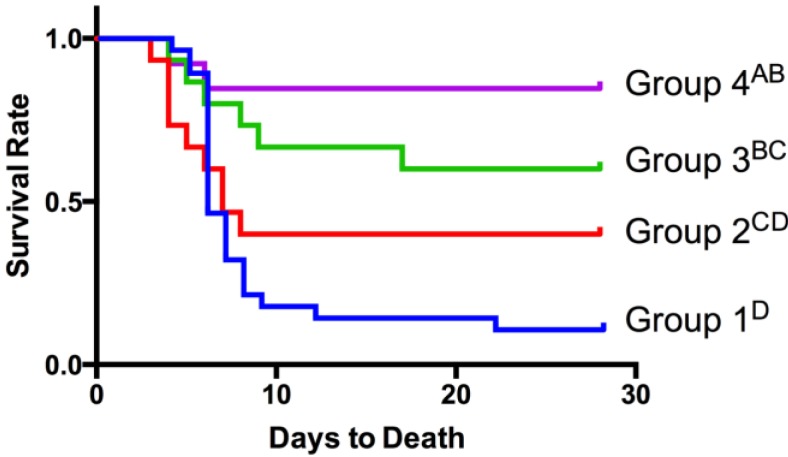
Kaplan Meier survival curves for black-tailed prairie dog groups that consumed placebo or vaccine-laden baits. Group 1 consumed placebo baits containing RCN-TK- and was challenged at 30 or 270 days post initial vaccination (dpv). The other 3 groups consumed baits containing RCN-F1/V307. Groups 2 and 3 were challenged at 30 and 270 dpv, respectively. Group 4 consumed baits a second time 30 days prior to challenge at 270 dpv. Survival curves that do not share a letter are significantly different (*p* < 0.05).

Survival rates were compared between all groups of PDs using Kaplan Meier survival analysis (Figure 3). As expected the two PD groups that received the empty vector only (RCN-TK-) and were challenged at 30 or 270 dpv had the lowest survival rates from plague challenge (13% and 8% respectively). As these two groups (group 1a and 1b in Table 5) were not significantly different (*p* > 0.05), they were combined for all further analyses.

**Table 5:** Survival of prairie dogs that voluntarily consumed baits containing RCN-F1/307 (with and without a boost) or RCN-TK- (placebo) and were challenged with *Yersinia pestis* at 30 and 270 days post initial vaccination (dpv). Risk ratios are calculated in relation to the group consuming placebo baits (RCN-TK-). Lower and upper 95% indicate 95% confidence intervals on the risk ratio.

Group	*n*	Bait Consumption		Challenge dpv	% Survival	Risk Ratio	Prob > Chisq	Lower 95%	Upper 95%
		Initial	Boost						
1a + 1b	28	RCN-TK-	-	30, 270	11
2	14	RCN-F1/V307	-	30	43	0.575	0.1603	0.241	1.232
3	15	RCN-F1/V307	-	270	60	0.308	0.0048	0.113	0.713
4	13	RCN-F1/V307	RCN-F1/V307	270	85	0.108	<0.0001	0.017	0.368

Forty-three % of PDs that consumed baits containing RCN-F1/V307 and were challenged with *Y. pestis* 30 dpv survived infection, but their risk ratio was not significantly different (*p* = 0.1603) from the placebo group (Table 5). In contrast, 60% of PDs that consumed baits containing RCN-F1/307 and were challenged at 270 dpv survived, and their risk ratio was significantly higher (*p* = 0.0048) than the placebo group. Even higher survival rates (85%) were observed in prairie dogs that consumed SPV baits a second time at 240 dpv and were challenged at 270 dpv. Their risk ratio was significantly higher (*p* < 0.0001) than both the placebo group and the group challenged at 30 dpv (Table 5).

Geometric mean antibody titers measured at 30, 180, and 270 dpv in PDs are presented in Table 6. Mean anti-F1 and anti-V titers increased significantly (*p* < 0.05) within 30 dpv. Within 180 dpv, titers to both antigens had declined, but they were still significantly different than control values. Antibody titers of animals that consumed baits a second time at 240 dpv increased again by 270 dpv. Antibody titers to anti-V were higher than anti-F1 titers, however none of the titers were very high, increasing from pre-vaccination titers at most by 1 log.

**Table 6:** Geometric mean anti-F1 and anti-V antibody titers ± standard error (SE) were compared between prairie dogs that consumed baits containing RCN-F1/V307 (with or without a boost) and RCN-TK- (placebo). Antibody titers were measured at 30, 180 and 270 days post initial vaccination (dpv).

Treatment	Mean Anti-F1 ± SE (*n*)*			Mean Anti-V ± SE (*n*)*		
	30 dpv	180 dpv	270 dpb	30 dpv	180 dpv	270 dpv
RCN-F1/V307	2.02 ± 0.08 ^A^ (42)	1.71 ± 0.07 ^A^ (28)	1.76 ± 0.12 ^AB^ (15)	2.20 ± 0.09 ^A^ (42)	1.95 ± 0.08 ^A^ (28)	1.96 ± 0.14 ^B^ (15)
RCN-F1/V307 with boost			1.97 ± 0.16 ^A^ (13)			2.67 ± 0.31 ^A^ (13)
RCN-TK-	1.60 ± 0.00 ^B^ (28)	1.60 ± 0.00 ^B^ (13)	1.60 ± 0.00 ^B^ (13)	1.60 ± 0.00 ^B^ (28)	1.60 ± 0.00 ^B^ (13)	1.60 ± 0.00 ^B^ (13)

* Values that do not share a letter within columns are significantly different at *p* < 0.05.

## 4. Discussion

Like other orthopoxviruses, RCN has proven to be an efficient vector for vaccine antigens, capable of delivering several antigens simultaneously. In this paper, we describe the construction of an RCN vectored vaccine expressing two protective *Y. pestis* antigens (designated RCN-F1/V307) and compare the efficacy of this dual antigen vaccine construct to the simultaneous administration of two single antigen vaccines (RCN-F1 + RCN-V307). The RCN-F1/V307 was found to be highly stable following *in vitro* serial passage in Vero cells and expressed both F1 and V307 antigens. In mice, RCN-F1/V307 was as effective at preventing plague as the simultaneous administration of RCN-F1 + RCN-V307. Although the dual antigen construct was a little slower in inducing protection than the combination vaccine, by 56 dpb, mice vaccinated with RCN-F1/V307 had similar survival rates (67%) as those vaccinated with RCN-F1 (50%) or RCN-F1 + RCN-V307 (33%). At least 50% of mice vaccinated with RCN-F1/V307 survived *Y. pestis* challenge doses as high as 8 × 10^5^ mouse LD_50_s. This protective efficacy is similar to our previous findings in mice vaccinated simultaneously with the two single antigen constructs [9].

Even more importantly, our findings demonstrated that RCN-F1/V307 can induce significant protection in orally vaccinated PDs, even after a single administration of the vaccine. Protective efficacy seems to improve with time (up to 60% at approximately 9 months post vaccination), although additional work is required to establish the kinetics of protection at both shorter (1–9 months) and longer times (>9 months) post vaccination. A booster vaccination via consumption of baits a second time provided even greater protection (85%) in PDs. This level of protection is very similar to results achieved in PDs (94%) that consumed baits with the combination vaccine (RCN/F1 + RCN/V307), with a 178 day interval between initial and booster vaccinations [6]. These results will help inform the design and evaluation of future field studies in wild PD colonies.

We further demonstrated that RCN-F1/V307 protects mice against an F1 negative strain of *Y. pestis.* Mice vaccinated with RCN-F1 alone succumbed to challenge with F1 negative *Y. pestis*, whereas, approximately half the mice that received either RCN-F1/V307 or simultaneous injections of RCN-F1 and RCN-V307 survived the same challenge. Both these groups of mice had high anti-V antibody titers. Although not tested yet, we hypothesize that orally vaccinated PDs would also be protected against an F1 negative *Y. pestis* strain.

Previous work showed that a truncated version of the *lcr*V gene (V307) was as effective as the entire V gene at eliciting a protective immune response in combination with F1 [9]. This V307 antigen presumably avoids some of the immunomodulatory properties associated with full V protein [13], including suppression of gamma interferon and tumor necrosis factor alpha *in vivo* [14,15]. However, vaccination with RCN-V307 alone was not very effective and resulted in the poorest survival rate upon plague challenge of all the groups. Administration of RCN-F1/V307 or the combination of RCN-F1 and RCN-V307 resulted in the highest rates of protection against plague in mice. This result supports our previous findings in mice that simultaneous vaccination with two antigens provides increased protection against plague challenges [6,9].

Interestingly, vaccination with the dual antigen vaccine resulted in different antibody responses compared to the combination vaccine. In mice, administration of RCN-F1/V307 elicited significantly higher antibody titers to V compared to the combination vaccine but lower titers to F1. *In vitro* results also demonstrated higher levels of V307 expression in the RCN-F1/V307 construct, which suggests that simultaneous expression of *Y. pestis* antigens may result in F1 interference. We have previously observed this phenomenon in the design of rRCN vaccines against plague and other pathogens [16]. It is possible that this viral interference can be overcome by using different viral promoters or stronger translation enhancer sequences. Despite the interference, in this study, RCN-F1/V307 expressed sufficient antigen levels to confer protection against both F1 positive and F1 negative strains.

Similar serologic results were obtained with PDs that consumed baits containing RCN-F1/V307, although titers were much lower relative to mice, likely due to the different routes of vaccine administration (oral *versus* i.m.). Unfortunately, laboratory mice are not susceptible to infection by RCN via the oral route [17] for better comparison. Anti-V titers were slightly higher in PDs than anti-F1 titers, and the levels of both were very similar to those reported in PDs that consumed baits with the combination vaccine [6]. Protection of PDs against plague in the absence of high IgG titers suggests other mechanisms of immunity may be playing a role in protection. Other poxvirus recombinants (vaccinia expressing the luciferase reporter gene, HIV env protein, or β-galactosidase) delivered orally to mice resulted in significant mucosal IgA responses and both local and systemic cellular immune responses [18]. Future studies will determine the role of mucosal and cellular immune responses in mice and PDs.

## 5. Conclusions

An ideal plague vaccine for wildlife would be safe, highly efficacious, stable, deliverable orally, easy to distribute to large numbers of animals in the field, and of relatively low cost to produce. Our dual antigen vaccine construct, as described here, meets all those requirements. Our data confirms that RCN-F1/V307 is stable, efficacious in protecting PDs in laboratory challenge studies, and can be delivered orally via baits. Consumption of baits a single time can be protective, but a second consumption provides even higher rates of protection, suggesting that booster vaccinations may be necessary to achieve full protection in the field. In this and previous studies, we exposed PDs to *Y. pestis* via the subcutaneous route to achieve a standardized challenge inoculum, but in the field, plague exposure occurs via multiple flea bites and possibly via other means. Because different routes of exposure could affect the outcome of vaccination, field studies will be needed to determine if SPV is efficacious in preventing plague in nature.
